# Atypical spinal cord infarction: A case report

**DOI:** 10.1097/MD.0000000000011058

**Published:** 2018-06-18

**Authors:** Koshi Ota, Ryo Iida, Kanna Ota, Masahide Sakaue, Shogo Takashima, Kohei Taniguchi, Masao Tomioka, Masahiko Nitta, Akira Takasu

**Affiliations:** Department of Emergency Medicine, Osaka Medical College, Osaka, Japan.

**Keywords:** abrupt onset of bilateral sensorimotor deficits, apparent diffusion coefficient, diffusion-weighted imaging, magnetic resonance imaging, spinal cord infarction

## Abstract

**Introduction:**

The abrupt onset of sensorimotor deficits is a neurologic emergency that requires immediate management. Acute spontaneous spinal cord infarction (SCI) is rare, but can cause the sudden onset of quadriplegia or quadriparesis. Magnetic resonance imaging (MRI) is an essential imaging modality to diagnose SCI.

**Case presentation:**

A 75-year-old man with a history of diabetes mellitus type 2, hypertension, and dyslipidemia was transferred to our facility for further workup of the sudden onset of quadriplegia. Diffusion-weighted contrast MRI (DWI) on hospital day 8 revealed hyperintense signals predominantly at the grey matter, and a contrast T2 signal abnormality with a decreased apparent diffusion coefficient (ADC). Steroid pulse therapy was initiated because myelitis could not be completely ruled out, but this did not improve the neurological deficits. Spontaneous SCI was finally diagnosed as an exclusion diagnosis. Symptoms were gradually recovered with rehabilitation, and he was transferred to a rehabilitation facility on hospital day 40.

**Conclusion:**

MRI with DWI of the spine should be considered for an early diagnosis of SCI. A combination of DWI with ADC maps is recommended to distinguish SCI from other differential disorders.

## 1 Introduction

The abrupt onset of bilateral sensorimotor deficits is a neurologic emergency that requires immediate management. Several etiologies, including acute spinal cord infarction (SCI), can cause the sudden onset of quadriplegia or quadriparesis.^[1]^ The neurological symptoms brought about by vascular disruption caused by an ischemic lesion are important to consider from an anatomical viewpoint. The anterior spinal artery is distributed to the anterior two thirds of the spinal cord including the anterior horns of the gray matter, the spinothalamic tract, and the corticospinal tract, and thus can be involved in the symptoms of acute SCI.^[2]^ Weakness and sensory loss with spared proprioception (body position in space and vibratory sense) are the common clinical presentations of SCI of the anterior spinal artery.^[3]^ However, several atypical SCI presentations do not fit the anatomically defined spinal blood distribution.^[4,5]^ Magnetic resonance imaging (MRI) is an essential imaging modality to rule out misdiagnoses of SCI such as compressive myelopathy.^[4–6]^ Here, we describe a patient with SCI that was difficult to diagnose because of atypical manifestations and ambiguous MRI findings. We obtained informed consent from the patient and his wife for reporting this case.

### 1.1 Case report

**Figure 1:**
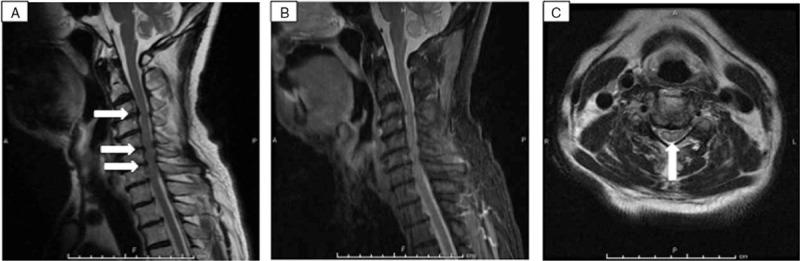
Magnetic resonance imaging (MRI) findings upon admission. A, Sagittal T2-weighted (3000/106.10 [TR/TE]) image shows degenerative changes at C3/4, C5/6, and C6/7 (arrow). B, Sagittal short T1 inversion recovery (STIR; 3500/16.91 [TR/TE]) image shows the same findings as in (A). C, Axial T2-weighted (3866.67/89.98 [TR/TE]) image shows hyperintense signal with involvement of grey matter and adjacent central white matter at C4/5 (arrow).

**Figure 2:**
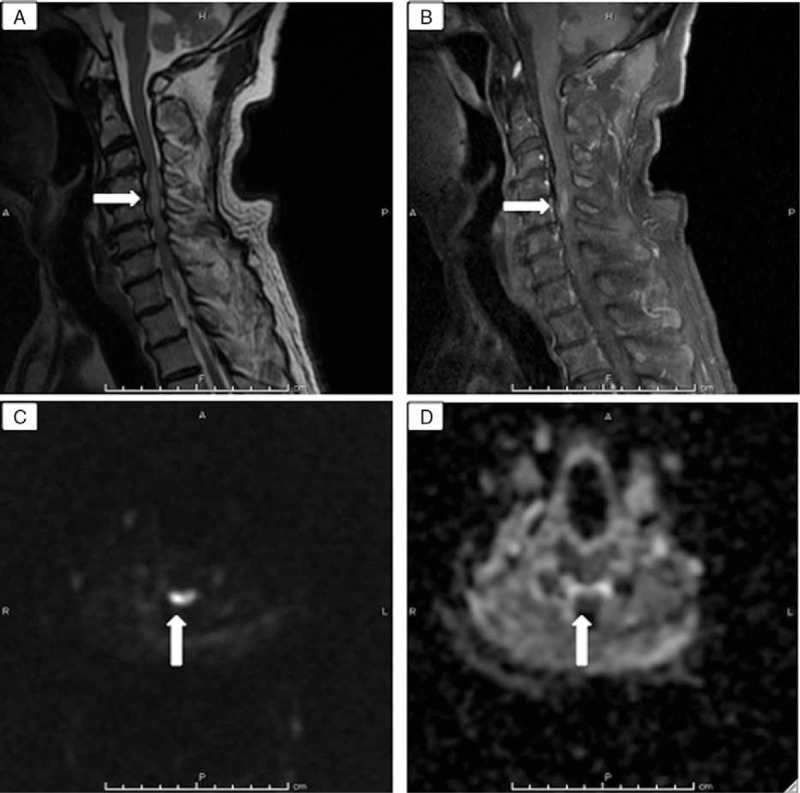
Magnetic resonance imaging (MRI) findings on hospital day 8. High signal intensity at C4/5 (arrows) on (A) sagittal contrast-enhanced T2-weighted (3000/99.21 [TR/TE]) image, (B) T1-weighted (516.67/11.96 [TR/TE]) image, and (C) axial diffusion weighted imaging (DWI; 4775/68.30 [TR/TE]) image. D, Axial ADC (4775/68.30 [TR/TE]) map shows a dark area at C4/5 (arrow).

**Figure 3:**
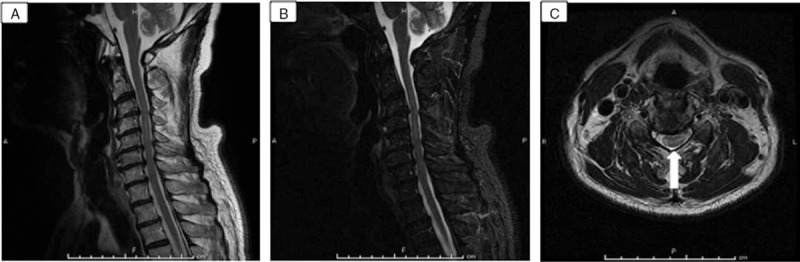
Magnetic resonance imaging (MRI) findings on hospital day 20. A, Sagittal T2-weighted (3516.67/87.65 [TR/TE]) and (B) short T1 inversion recovery (STIR; 4100/85.27 [TR/TE]) images. C, Hyperintense signal persists at C4/5 on axial T2-weighted (3500/109.20 [TR/TE]) image (arrow).

A 75-year-old man with a history of diabetes mellitus type 2, hypertension, and dyslipidemia presented to a local community hospital with neck and back pain that had persisted for 4 days. During a medical examination, he felt the abrupt onset of weakness in the upper and lower extremities and suddenly became unable to walk. Computed tomography (CT) of the brain did not reveal any abnormalities and the patient was transferred to our facility for further workup of the sudden quadriplegia. His vital signs upon arrival at the emergency room were as follows: body temperature, 35.9°C; pulse rate, 64 beats per minute; respiratory rate, 21 breaths per minute; blood pressure, 187/87 mm Hg; and oxygen saturation, 97% on ambient air. His speech, cognition, and cranial nerve function were normal. Manual muscle tests (MMTs) showed left-right symmetrical findings (Table 1). The passive range of motion in all extremities was complete without pain or spasticity. Reflexes were bilaterally overactive in the biceps, triceps, and brachioradialis, especially in the patellar and Achilles tendons, and Babinski sign was also present bilaterally. A rectal examination revealed normal function. Proprioception (body position in space and vibratory sense) was preserved, but pain sensation was lost below the T4 level on the right side. Urinary incontinence also developed and a urethral catheter was inserted. Other than mild calcification of the aorta, whole-body CT findings including the cervical, thoracic, and lumbar spine were normal. Initial MRI revealed abnormal T2 signals in the cervical spine from C4 to C5 with cord compression from C3 to C7 (Fig. 1). An orthopedic surgeon was consulted under consideration of a diagnosis of cervical spondylotic myelopathy (CSM). Repeat MMT findings showed a left-right asymmetrical change (Table 1). The findings of cerebrospinal fluid (CSF) obtained via lumbar puncture on the day after admission were: white blood cells, 2 cells/mm^3^; red blood cells, 25 cells/mm^3^; total protein, 104.7 (normal 15–45) mg/dL; and glucose, 83 (normal (70–110) mg/dL. Hematological and biochemical findings were mostly within normal ranges except for mildly elevated creatinine, 1.24 mg/dL; blood sugar, 181 mg/dL; triglyceride, 173 mg/dL; and hemoglobin A1c, 6.4%. Antihuman T-lymphotropic virus type I (HTLV-I) antibody was positive in both blood and CSF specimens. No specific treatment except rehabilitation was performed until neurology consult because etiology was unknown. Diffusion-weighted contrast MRI (DWI) on hospital day 8 after a neurology consult revealed hyperintense signals predominantly at the grey matter, and a contrast T2 signal abnormality with a decreased apparent diffusion coefficient (ADC) (Fig. 2). We started the patient on steroid pulse therapy because myelitis could not be completely ruled out, but this did not improve the neurological deficits. SCI was finally diagnosed as an exclusion diagnosis. The MRI findings on hospital day 20 were the same as the initial findings (Fig. 3). The patient started to gradually recover even before the administration of steroid pulse therapy, although he needed a walking aid. He was transferred to a rehabilitation facility on hospital day 40.

**Table 1:**
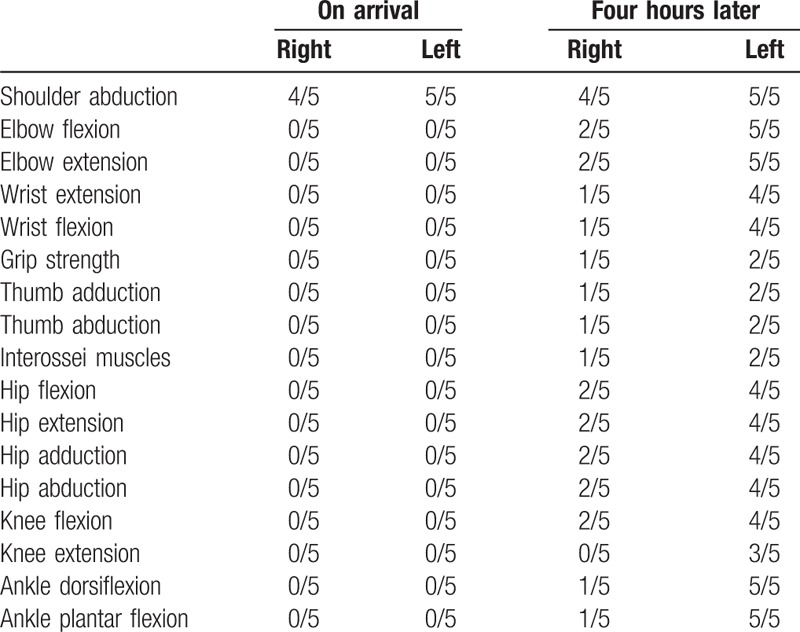
Findings of manual muscle testing.

## 2 Discussion

SCI is rare and the diagnosis is challenging in the face of atypical presentations. Our patient developed quadriplegia within 1 minute and initial T2-weighted MRI revealed degenerative changes in the spinal cord that indicated CSM. Therefore he was referred to an orthopedic surgeon. CSM is the most common cause of progressive disability; it impairs the quality of life among elderly persons due to spinal dysfunction, and symptoms usually begin insidiously, starting with gait impairment.^[7]^ Cord compression on MRI was compatible with a diagnosis of CSM in our patient. In addition, the course was sudden, without trauma, and clearly different from that of SCI. We also considered HTLV-I-associated myelopathy, which also has insidious clinical features.^[8]^

Guillain-Barré syndrome (GBS) should be taken into account for a differential diagnosis of SCI, because it is characterized by rapid symmetrical muscle weakness and elevated CSF protein with a normal white blood cell count. The albuminocytological dissociation in CSF from our patient was compatible with GBS, but the hyper-reflexive tendons did not seem to correlate with this diagnosis. Acute myelitis from neuromyelitis optica is yet another differential diagnosis, which is difficult to differentiate from SCI using MRI.^[4]^ Neuromyelitis optica tends to develop around lesions of the cervicomedullary and cervicothoracic junctions, where SCI is rare due to an abundant collateral blood supply.^[4]^ In addition, the steroid pulse therapy that might have been beneficial against myelitis did not improve the symptoms in our patient.

Several etiological factors such as hypertension, diabetes mellitus, dyslipidemia, atherosclerosis, and degenerative disease of the spine might have been associated with SCI in our patient.^[1,3]^ Sudden quadriplegia is the most important feature of SCI, although hyperreflexia and pathological reflexes at the initial examination are unlikely during the acute SCI phase. A combination of DWI with ADC maps is recommended to distinguish SCI from myelitis and demyelinating disorders, and conclude a diagnosis.^[5,9,10]^ SCI usually appears on T2-weighted MRI as abnormally hyperintense signals, predominantly in the grey matter, and DWI of our patient identified a hyperintense signal in areas of T2 signal abnormalities with a decreased ADC value. There have not been standardized guidelines for the treatment of SCI.^[1]^ The outcome is variable and determined by the degree of SCI and subsequent rehabilitation. In general, long-term outcome of SCI is better than cerebral infarction.^[11]^

In conclusion, although the initial clinical presentation of SCI is variable and nonspecific, the abrupt onset of weakness and sensory symptoms should be considered as SCI. An early diagnosis of SCI is important, and MRI with DWI of the spine should proceed as soon as possible to ensure appropriate management.

## Author contributions

**Conceptualization:** Koshi Ota.

**Resources:** Kanna Ota, Ryo Iida, Masahide Sakaue, Shogo Takashima, Masao Tomioka.

**Supervision:** Koshi Ota, Akira Takasu.

**Writing – original draft:** Koshi Ota, Akira Takasu.

**Writing – review and editing:** Koshi Ota, Kanna Ota, Kohei Taniguchi, Masahiko Nitta, Akira Takasu.

## References

[1] Novy J Carruzzo A Maeder P Spinal cord ischemia: clinical and imaging patterns, pathogenesis, and outcomes in 27 patients. Arch Neurol 2006;63:1113–20. 16908737 10.1001/archneur.63.8.1113

[2] Kumral E Polat F Güllüoglu H Spinal ischaemic stroke: clinical and radiological findings and short-term outcome. Eur J Neurol 2011;18:232–9. 20402756 10.1111/j.1468-1331.2010.02994.x

[3] Masson C Pruvo JP Meder JF Spinal cord infarction: clinical and magnetic resonance imaging findings and short term outcome. J Neurol Neurosurg Psychiatry 2004;75:1431–5. 15377691 10.1136/jnnp.2003.031724 PMC1738740

[4] Kister I Johnson E Raz E Specific MRI findings help distinguish acute transverse myelitis of neuromyelitis optica from spinal cord infarction. Mult Scler Relat Disord 2016;9:62–7. 27645347 10.1016/j.msard.2016.04.005

[5] Küker W Weller M Klose U Diffusion-weighted MRI of spinal cord infarction. J Neurol 2004;251:818–24. 15258783 10.1007/s00415-004-0434-z

[6] Weidauer S Nichtweiß M Hattingen E Spinal cord ischemia: aetiology, clinical syndromes and imaging features. Neuroradiology 2015;57:241–57. 25398656 10.1007/s00234-014-1464-6

[7] Baron EM Young WF Cervical spondylotic myelopathy: a brief review of its pathophysiology, clinical course, and diagnosis. Neurosurgery 2007;60 (1 suppl 1):S35–41. 17204884 10.1227/01.NEU.0000215383.64386.82

[8] Gotuzzo E Cabrera J Deza L Clinical characteristics of patients in peru with human T cell lymphotropic virus type 1–associated tropical spastic paraparesis. Clin Infect Dis 2004;39:939–44. 15472843 10.1086/423957

[9] Loher TJ Bassetti CL Lovblad KO Diffusion-weighted MRI in acute spinal cord ischaemia. Neuroradiology 2003;45:557–61. 12830338 10.1007/s00234-003-1023-z

[10] Takeshita S Ogata T Mera H Time course of diffusion weighted image and apparent diffusion coefficient in acute spinal cord infarction: a case report and review of the literature. Rinsho Shinkeigaku 2016;56:352–5. 27098903 10.5692/clinicalneurol.cn-000858

[11] Hanson SR Romi F Rekand T Long-term outcome after spinal cord infarctions. Acta Neurol Scand 2015;131:253–7. 25346212 10.1111/ane.12343

